# The Integrated Probiotic Database: a genomic compendium of bifidobacterial health-promoting strains

**DOI:** 10.20517/mrr.2021.13

**Published:** 2022-02-28

**Authors:** Chiara Tarracchini, Martina Viglioli, Gabriele Andrea Lugli, Leonardo Mancabelli, Federico Fontana, Giulia Alessandri, Francesca Turroni, Marco Ventura, Christian Milani

**Affiliations:** ^1^Laboratory of Probiogenomics, Department of Chemistry, Life Sciences, and Environmental Sustainability, University of Parma, Parco Area delle Scienze 11a, Parma 43124, Italy.; ^2^GenProbio Srl, Via delle Scienze, 11/A, Parma 43100, Italy.; ^3^Microbiome Research Hub, University of Parma, Parco Area delle Scienze 11a, Parma 43124, Italy.

**Keywords:** Bifidobacterium, bifidobacterium longum, bifidobacterium infants, bifidobacterium bifidum, bifidobacterium breve, bifidobacterium animalis, genomics

## Abstract

**Background: **The World Health Organization defines probiotics as “live microorganisms, which when administered in adequate amounts confer a health benefit on the host”. In this framework, probiotic strains should be regarded as safe for human and animal consumption, i.e., they should possess the GRAS (generally recognized as safe) status, notified by the local authorities. Consistently, strains of selected *Bifidobacterium* species are extensively used as probiotic agents to prevent and ameliorate a broad spectrum of human and/or animal gastrointestinal disorders. Even though probiotic properties are often genus- or species-associated, strain-level differences in the genetic features conferring individual probiotic properties to commercialized bifidobacterial strains have not been investigated in detail.

**Methods:** In this study, we built a genomic database named Integrated Probiotic DataBase (IPDB), whose first iteration consists of common bifidobacterial strains used in probiotic products for which public genome sequences were available, such as members of *B. longum* subsp. *longum*, *B. longum* subsp. *infantis*, *B. bifidum*, *B. breve*, and *B. animalis* subsp. *lactis *taxa. Furthermore, the IPDB was exploited to perform comparative genome analyses focused on genetic factors conferring structural, functional, and chemical features predicted to be involved in microbe-host and microbe-microbe interactions.

**Results and conclusion:** Our analyses revealed strain-level genetic differences, underlining the importance of inspecting the strain-specific and outcome-specific efficacy of probiotics. In this context, IPDB represents a valuable resource for obtaining genetic information of well-established bifidobacterial probiotic strains.

## INTRODUCTION

The widely accepted definition of probiotics as “live microorganisms that when administered in adequate amounts confer a beneficial health effect on the host” was given by the Food and Agriculture Organization of the United Nations and the World Health Organization in 2001^[1]^. Such health beneficial effects may include participation in complex carbohydrates digestion; vitamins, amino acids, and short-chain fatty acids production; antagonistic activity against intestinal bacterial pathogens; and immune system modulation^[2]^. Nevertheless, to be considered a valuable probiotic, microbial strains must also meet specific criteria, including surviving passage through the upper digestive tract due to low pH and bile salts resistance, the ability to adhere to the human gut mucosa, and the ability to colonize the human intestine. In addition, a probiotic strain must be safe for human consumption^[3]^.

Most microorganisms recognized to date as probiotics are Gram-positive bacteria, including members of *Enterococcus*, *Streptococcus*, *Lactobacillus*, and *Bifidobacterium* genera^[4]^. In particular, members of this latter genus are among the main microorganisms used as probiotics in the global market^[5,6]^. Indeed, several members of the *Bifidobacterium* genus recognized as GRAS (generally recognized as safe) are widely and extensively included as live components in commercial probiotic products, either alone or in multi-strain formulations^[7,8]^. In this context, despite bifidobacterial probiotic strains and related commercial products being accompanied by specific health-promoting claims, comparative analyses focusing on the genetic factors related to probiotic features are still lacking.

In this study, we built a genome database of the bifidobacterial strains employed in approved commercial probiotic dietary products named the Integrated Probiotic DataBase (IPDB). In detail, 34 genomes corresponding to *B. longum* subsp. *longum*, *B. longum* subsp. *infantis*, *B. bifidum*, *B. breve*, and *B. animalis* subsp. *lactis* commercial probiotics were retrieved from public repositories based on extensive literature screening and processed through an optimized bioinformatics pipeline for genes prediction and functional annotation. Further, we carried out a comparative genome analysis to identify the main shared and unique genetic features related to colonization, survival, and persistence in the gastrointestinal tract. Besides, the presence of intrinsic antimicrobial resistance (AMR) was also assessed since it could be valuable to prevent/reduce gut microbiota disorders during antibiotic treatments.

## METHODS

### Genome sequences of bifidobacterial commercial strains

In accordance with scientific literature surveys, publicly available chromosomal sequences of 34 bifidobacterial strains used in commercial dietary probiotic products were retrieved from the National Center for Biotechnology Information (NCBI) public database [Table 1]. To ensure a consistent genomic analysis, open reading frames (ORFs) from each bifidobacterial genome sequence were re-predicted and annotated using the most recent release of the MEGAnnotator pipeline^[9]^. In detail, contigs greater than 1000 bp were employed to predict protein-encoding ORFs through Prodigal v2.0 (Linux command line “./prodigal -f gff -a [protein_translation_to_selected_file] -i [input_filename.fasta] -o [output_filename]”)^[10]^. Predicted ORFs were then functionally annotated using RAPSearch2 (reduced alphabet-based protein similarity search) (cutoff e-value of 1 × 10^-5^ and minimum alignment length 20) employing the NCBI reference sequences (RefSeq) database^[11]^ together with hidden Markov model profile (HMM) searches (http://hmmer.org/) against the manually curated Pfam-A database (cutoff e-value of 1 × 10^-10^). Then, tRNA genes were detected through tRNAscan-SE v1.4^[12]^, while rRNA genes were identified using RNAmmer v1.2^[13]^.

**Table 1:** Publicly available bifidobacterial commercial probiotic strains included in the IPDB

	**Strain name**	**Assembly No.**	**Genome status**	**Genome size (Mb)**	**GC content (%)**	**No. of CDS**
***B. animalis*** ** spp. *lactis***	BB-12	GCA_000025245.2	Complete	1.94	60.5	1570
	BLC1	GCA_000224965.2	Complete	1.94	60.5	1565
	B420	GCA_000277325.1	Complete	1.94	60.5	1568
	BS 01	GCA_018408975.1	Draft	1.93	60.5	1728
	HN019	GCA_003606305.1	Complete	1.94	60.5	1567
	BS 05	GCA_018408985.1	Draft	2.09	60.6	1720
	MB 2409	GCA_018409015.1	Draft	1.97	60.4	1685
	Bl-04	GCA_000022705.1	Complete	1.94	60.5	1568
	Bi-07	GCA_000277345.1	Complete	1.94	60.5	1566
	ADO 11	GCA_000021425.1	Complete	1.93	60.5	1582
	BL-G101	GCA_017963615.1	Draft	1.92	60.5	1568
	BL3	GCA_002220485.1	Complete	1.94	60.5	1574
	BPL1 (CECT 8145)	GCA_000612705.1	Draft	1.96	60.4	1633
***B. longum*** ** spp. *infantis***	Bi-26	GCA_004919065.2	Complete	2.61	59.3	2237
	UBBI-01	GCA_004803425.1	Draft	2.73	59.4	2462
	35624	GCA_001719085.1	Complete	2.26	60	1827
	EVC001	GCA_902167885.1	Complete	2.83	59.9	2567
***B. longum*** ** spp. *longum***	BORI	GCA_003342655.1	Complete	2.31	59.9	1831
	W11	GCA_001940535.1	Draft	2.33	59.9	1886
	BL 03	GCA_018409185.1	Draft	2.35	60	2010
	DLBL 07	GCA_018409165.1	Draft	2.37	59.9	1992
	DLBL 09	GCA_018408965.1	Draft	2.37	59.8	1989
	CECT 7347 (ES1)	GCA_001050555.1	Draft	2.33	60	2019
	BB536	BAA-999 (ATCC site)	Complete	2.42	59.9	2023
	JDM301	GCA_000092325.1	Complete	2.48	59.8	2024
	KACC 91563	GCA_000219455.1	Complete	2.40	59.8	1952
	CECT 7894	GCA_016634435.1	Draft	2.29	59.9	1873
***B. bifidum***	PRL2010	GCA_000165905.1	Complete	2.21	62.7	1830
	BGN4	GCA_000265095.1	Complete	2.22	62.6	1792
	BF3	GCA_001281345.1	Complete	2.21	62.6	1782
	ATCC 29521	GCA_000466525.1	Draft	2.2	62.7	1846
***B. breve***	BR03	GCA_004319685.1	Draft	2.27	58.6	1871
	BB02	GCA_002914865.1	Draft	2.32	58.8	1983
	UBBR-01	GCA_004802595.1	Draft	2.33	58.7	2043

IPDB: Integrated Probiotic DataBase.

### Comparative genomic analysis

All 34 genome sequences of *Bifidobacterium* members were employed for a pan-genome analysis using the Pangenome Analysis Pipeline (PGAP) v1.1 (http://pgap.sf.net)^[14]^. The predicted proteome of each bifidobacterial genome was classified into functional gene clusters through the gene family (GF) method, consisting of pairwise protein-similarity search using blast software v2.2.28+ (cutoff e-value of 1 × 10^-10^ and exhibiting at least 50 % identity across at least 80 % of both protein sequences). The obtained data were used to assign proteins to so-called clusters of orthologous groups (COGs) employing MCL (graph theory-based Markov clustering algorithm)^[15]^. A pan-genome profile was then built using a presence/absence matrix encompassing all COGs identified in the analyzed genomes (Linux command line “./PGAP.pl --strains [input_strain_list] --input input_path/ --output output_path/ --thread 20 --identity 0.5 --coverage 0.8 --cluster --method GF --evolution --pangenome”). Subsequently, the core genome of commercial bifidobacterial strains was obtained by selecting the protein families shared between all genomes, while truly unique genes (TUGs) of a given genome were identified based on those protein families that are not present in other bifidobacterial chromosomes. Functional annotation of each TUG arsenal was accomplished employing the eggNOG database^[16]^. Each pairwise average nucleotide identity (ANI) was calculated using the program fastANI^[17]^.

### Phylogenomic analysis

To disentangle the phylogenetic relationships between the 34 collected bifidobacterial probiotic strains, the concatenated sequence of amino acids belonging to the core genome of each bifidobacterial strain was aligned using the MAFFT software^[18]^. The resulting phylogenetic tree was built using the neighbor-joining method through the ClustalW v2.1 program^[19]^, and the graphical viewer of phylogenetic trees FigTree v1.4 (http://tree.bio.ed.ac.uk/software/figtree/) was used to its visual representation.

### Glycobiome prediction and identification of genes conferring antimicrobial resistance

The genome sequences of the publicly available 34 bifidobacterial probiotic strains were subjected to assessment of genes encoding for glycosyl hydrolase (GH), glycosyl transferase (GT), and polysaccharides lyase (PL) enzymes through sequence similarity search in the carbohydrate-active enzyme (CAZy) database^[20]^ using HMMER v3.3^[21]^ (cutoff e-value of 1 × 10^-15^) and BLASTP analysis^[22]^ (cutoff e-value of 1 × 10^-10^).

The proteome of each bifidobacterial probiotics genome was also screened for the presence of bacterial antimicrobial resistance based on sequence similarity to genes classified in the Comprehensive Antibiotic Resistance Database^[23]^ (BLASTP cutoff e-value of 1 × 10^-5^). Outcomes were then manually validated to eliminate possible false positives. Moreover, the Transporter Classification DataBase (TCDB)^[24] ^was employed to assess the putative transporter specificity.

### Identification of sortase-dependent pilus-encoding loci, and bacteriocins-encoding genes

Sortase-dependent (SD) pilus-encoding loci (type I and II pili) were identified through homology search tool RAPsearch (cutoff E value of 1 × 10^-5^ with minimal alignment length 20)^[25]^ exploiting the custom sortase-dependent pilus genes database previously built^[26]^. Then, a detailed manual inspection was performed to identify complete pilus gene clusters.

Likewise, bacteriocin-encoding genes were detected using RAPsearch analysis (cutoff E value of 1 × 10^-5^ with minimal alignment length 20) employing the BAGEL4 database^[27]^.

### Assessment the genetic background for exopolysaccharides, virulence, and bile salt hydrolases production

To identify the loci encoding exopolysaccharides (EPSs), the protein sequences of well-known priming glycosyltransferases (pGTFs) were retrieved from NCBI database and were used to inspect the 34 bifidobacterial genome sequences. Subsequently, for each bifidobacterial chromosome, the genomic regions flanking the putative pGTF were investigated to identify EPS-encoding key genes (such as glycosyltransferases, flippases, ABC transporters, and carbohydrate precursor biosynthesis/modification enzymes). The presence of putative virulence genes and bile salt hydrolases were identified through sequence similarity (homology) search in the Virulence Factor Database^[28]^ and in the protein sequence NCBI database, respectively (cutoff E value of 1 × 10^-5^). Thus, the resulting hits were manually inspected to remove false positives.

### Statistical analyses

All statistical analyses were computed using SPSS software (www.ibm.com/software/it/analytics/spss/).

## RESULTS AND DISCUSSION

### The IPDB

Bifidobacterial strain names from labels of commercially available probiotic products were identified based on comprehensive scientific literature research, and all associated publicly available genomes (complete and draft) were retrieved from NCBI [Table 1]. As a result, we collected a total of 34 bifidobacterial probiotics, including 4 *B. longum* subsp. *infantis*, 10 *B. longum* subsp. *longum*, 4 *B. bifidum*, 3 *B. breve*, and 13 *B. animalis* subsp. *lactis* chromosomes sequences constituting the IPDB in its first iteration. Notably, to ensure consistency in the gene prediction, all bifidobacterial genomes used in this study were re-annotated using the MEGAnnotator pipeline as described in the Methods Section^[9]^. Subsequently, the 34 bifidobacterial commercial probiotic genomes were employed to perform a comparative genome analysis to identify peculiar genetic traits possibly involved in intestinal colonization and host-microbe interaction.

All the re-annotated genome sequences, along with strain-specific functional details and information concerning the comparative analysis results, are included in the newly developed IPDB available at http://probiogenomics.unipr.it/cmu/ (direct download at http://probiogenomics.unipr.it/files/Pro-biotic_Bifidobacteria_DataBase.zip). Note that IPDB will be expanded to include the genomes of non-bifidobacterial commercialized probiotic strains in the near future.

### General genome features of the bifidobacterial strains encompassed in the IPDB

According to the genome prediction and annotation processes, we identified a number of predicted ORFs ranging from 2567 for *B. longum* subsp. *infantis* EVC001 to 1565 for *B. animalis* subsp. *lactis* BLC1 [Table 1]. As previously reported, *B. longum* subsp. *infantis* showed the largest genomes among the probiotic collection, ranging between 2.83 and 2.61 Mb^[29]^, while *B. animalis* subsp. *lactis* resulted in the taxon with the smallest genome sizes (average of 1.95 Mb).

Notably, the ANI investigation highlighted a higher degree of genome identity among the 13 strains belonging to the *B. animalis* subsp. *lactis* species used as probiotics (average of 99.8%), compared to all the other considered (sub)species (average of 98.1%) [Supplementary Table 1]. Although the high degrees of synteny and sequence homology between members of this taxon is well-known (ANI ~99.7%)^[30]^, 53% of the *B. animalis* subsp. *lactis* strains showed ANI ≥ 99.99%, indicating that, presumably, identical strains have been effectively deposited and commercialized with different strain names. Moreover, according to the ANI analysis, the strain *B. longum* subsp. *longum* 35624, previously misclassified as a member of the *B. longum* subsp. *infantis* taxon, is still promoted commercially with an incorrect classification [Supplementary Table 1].

### Overview of the commercial probiotics pan-genome

**Figure 1:**
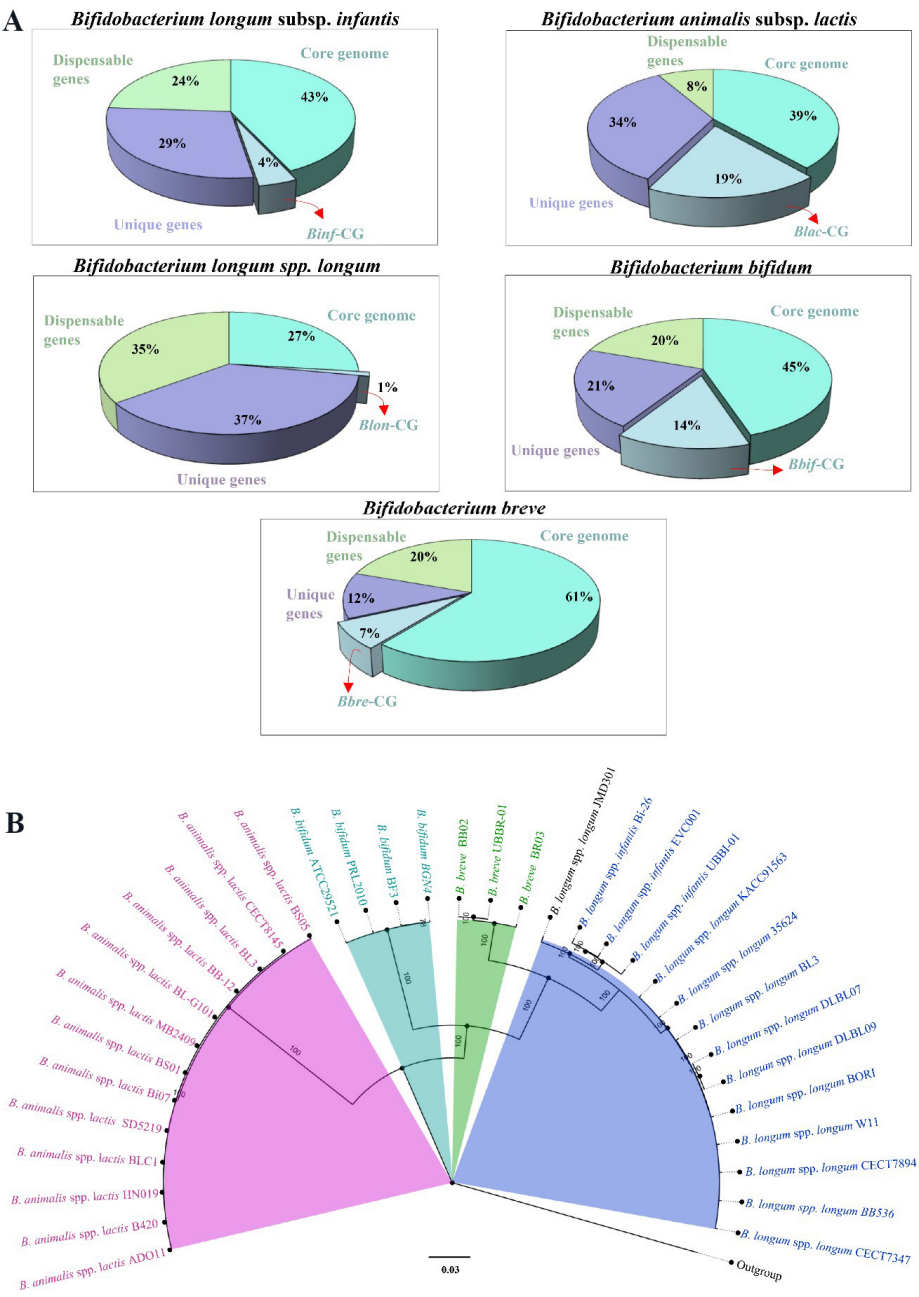
Pangenome of the five bifidobacterial (sub)species and phylogenetic relationships reconstruction. (A) The five (sub)species-specific pangenomes profiles. The core gene pools characterizing each bifidobacterial (sub)species, i.e., *Binf*-CG, *Blon*-CG, *Bbif*-CG, *Bbre*-CG, and *Blac*-CG, are highlighted as part of each core genome. (B) The phylogenomic tree based on the BPBs-CG describing the phylogenetic relationships among the 34 collected bifidobacterial probiotics. Each (sub)species-based cluster is highlighted with a different color.

The genome sequences of the 34 bifidobacterial probiotic strains were used to predict five (sub)species-specific pan-genome profiles by classifying each strain-specific proteome into protein families named COGs [Figure 1A]. Combining the obtained five (sub)species-specific pangenomes, we identified the core genome of the bifidobacterial probiotics (BPBs-CG) by taking into account a total of 657 COGs shared by all collected bifidobacterial (sub)species [Supplementary Table 2]. Similarly, five (sub)species-specific core genomes were obtained considering the COGs shared by all the strains belonging to a given sub(species) while being absent in the others. Accordingly, these latter were characterized by 150 *B. longum* subsp. *infantis* (*Binf*-CG), 90 *B. longum* subsp. *longum* (*Blon*-CG), 343 *B. bifidum (Bbif*-CG*)*, 169 *B. breve* (*Bbre*-CG), and 445 *B. animalis* subsp. *lactis* (*Blac*-CG) (sub)species-specific core genes (SSCore genes) [Figure 1A]. Notably, *B. longum* subsp. *longum* showed the fewest SSCore genes (ANOVA *P*-value < 0.05), suggesting that the evolutionary dynamics of this taxon have not led to achieving substantially unique genetic traits, while, in contrast, the phylogenetically correlated subspecies *B. longum* subsp. *infantis* showed a marked SSCore comparable to the *B. breve* species [Figure 1A and B]. Conversely, the relatively high number of SSCore genes of *Blac*-CG and *Bbif*-CG could reflect the evolutionary distance between *B. animalis* subsp. *lactis* and *B. bifidum* with respect to the other taxa included in this study, as pointed out by the phylogenetic reconstruction based on BPBs-CG [Figure 1A and B].

The pan-genome analysis also revealed the strain-specific genes repertoire, i.e., TUGs, highlighting a variable number of TUGs ranging from 403 to 12 (average of 122.6 TUGs per genome) [Figure 1A and Supplementary Table 2]. Notably, based on eggNOG analysis, bifidobacterial TUG arsenals included an average of 38.3% of genes with general or unknown function (R/S); an average of 16.5%, 13%, and 9% of genes predicted to be involved in DNA replication (M), carbohydrate (G), and amino acid (E) metabolisms, respectively; and the remaining 23.2% were related to cell wall/membrane biogenesis (M), defense mechanism (V), translation (J), transcription (K), and inorganic ion transport (P) [Supplementary Table 2]. Interestingly, *B. longum* subsp. *infantis* showed the highest number of TUGs (average of 322) [Supplementary Table 2]. This observation indicated peculiar features that may characterize the *B. longum* subsp. *infantis* strains employed as commercial probiotics^[29,31]^. Indeed, the relatively high degree of *B. longum *subsp. *infantis* genotype variation could be associated with the high rate of horizontal gene transfer events previously observed within this taxon^[29]^. In contrast, *B. animalis* subsp. *lactis* exhibited the fewest TUGs (average of 62.7) [Supplementary Table 2], corroborating the limited genetic variability among members of this taxon^[30]^, as evidenced by the abovementioned ANI analysis.

### Distribution of host-derived glycans metabolizing capabilities providing probiotic properties

Probiotic strains can metabolize the complex dietary carbohydrates that cannot be processed by host enzymes through the production of specific GHs, enhancing digestion and conferring health benefits to the host by releasing health-promoting compounds, such as Short-Chain Fatty Acids^[32]^. With the aim to investigate the differences in carbohydrate metabolizing capabilities of bifidobacterial probiotics, we explored the metabolic enzyme arsenal for complex carbohydrates, i.e., the glycobiome, catalyzing the breakdown of both dietary and host-derived carbohydrates. For each bifidobacterial probiotic strain, the complete glycobiome profile, including GHs, GTs, and PLs, is reported in Supplementary Table 3.

Based on the CAZy database^[20]^, we identified about 120 GHs per genome, corresponding to an average of 40.2 different GH families. In particular, 22 of the latter, including enzymes deputed to plant-derived carbohydrates metabolism as well as GH families active on glycosidic linkages of lactose, resulted to be included in the BPBs-CG, thus shared by all bifidobacterial probiotics [Supplementary Figure 1 and Supplementary Table 3].

Remarkably, additional enzymes degrading host-derived glycan structures (HMOs and intestinal mucin) such as GH101 (endo-α-N-acetylgalactosaminidase), GH20 (β-hexosaminidase), GH33 (sialidase), and GH129 (α-N-acetylgalactosaminidase) (www.cazy.org/) were detected in all bifidobacterial probiotics, except *B. animalis* subsp. *lactis*, and 27% of the *B. longum* subsp. *longum *strains. Consequently, these data highlight strain-dependent abilities of *B. longum* subsp. *longum* to digest HMOs-derived structures and, thus, to promote the absorption of nutrients during infant breastfeeding [Supplementary Figure 1 and Supplementary Table 3]. Furthermore, the GH29 family (α-L-fucosidases) was observed to be highly represented in *B. bifidum* and *B. longum *subsp. *infantis* chromosomes [Supplementary Figure 1 and Supplementary Table 3], while GH84 (exo-/endo-β-N-acetylglucosaminidases) and GH89 (extracellular soluble α-N-acetylglucosaminidases) were found exclusively within *Bbif*-CG, reflecting expanded metabolic capabilities toward host-derived glycan utilization of the abovementioned taxa, compared to the other *Bifidobacterium* probiotic (sub)species^[33-35]^.

Interestingly, members of the recently discovered GH136 family, which exert the role of extracellular lacto-N-biosidase^[36]^, beyond being shared by all *B. bifidum* probiotics, were found in 63% of those belonging to *B. longum* subsp. *longum *[Supplementary Figure 1 and Supplementary Table 3]. This observation might reveal a crucial survival strategy adopted by specific *B. longum* subsp. *longum* strains to increase their competitiveness in the infant gut ecosystem, although this subspecies is also adapted to utilize plant-derived oligosaccharides present in the adult diet^[37]^.

**Figure 2:**
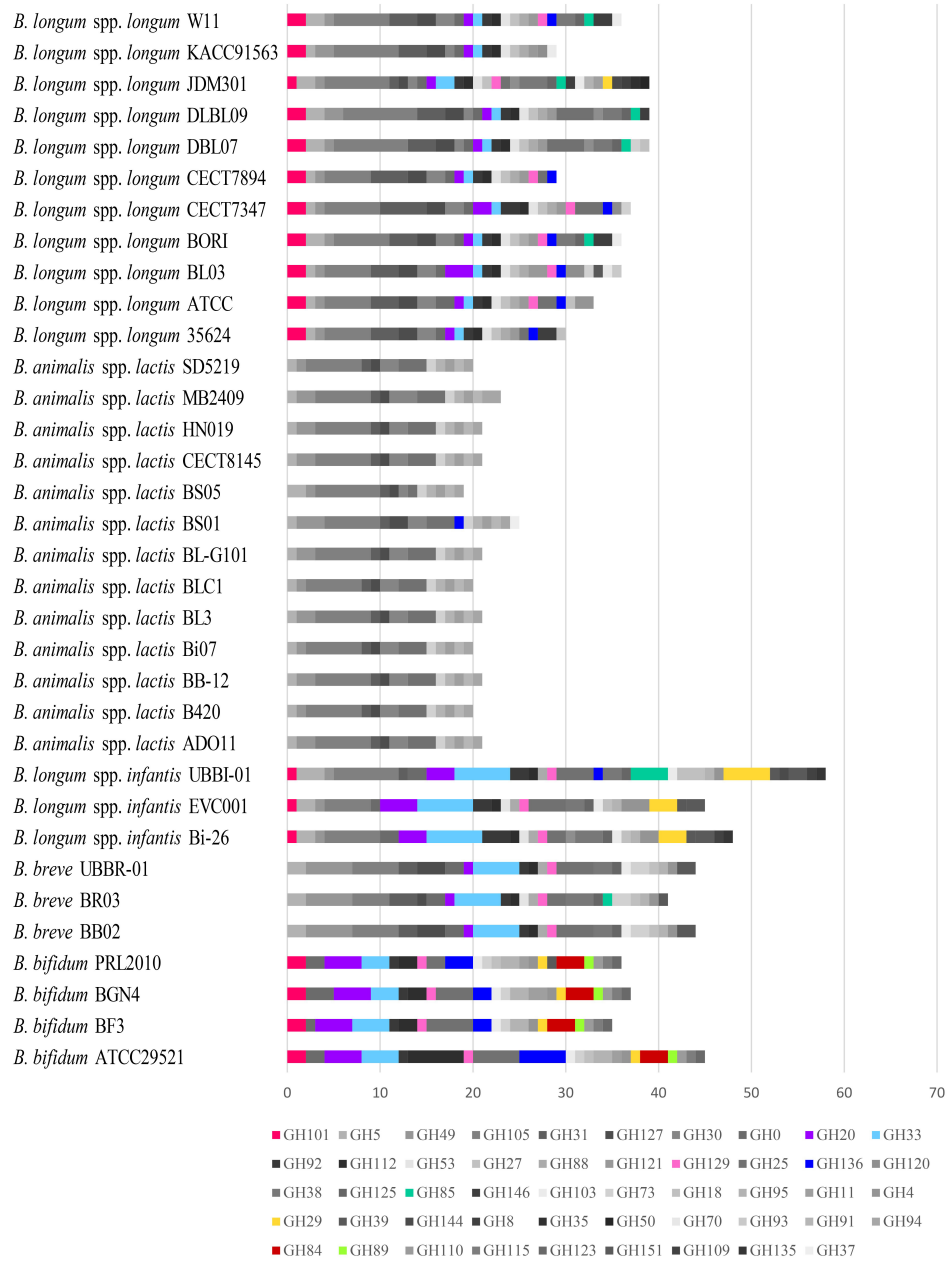
Accessory glycosyl hydrolase (GH) profiles of the bifidobacterial probiotics. For each bifidobacterial probiotic strain, the occurrence of the accessory GHs, i.e., GHs shared by a subset of the considered probiotic strains, are depicted through a bar-plot graph. The accessory GH families active on the host-derived glycans mentioned in the text are highlighted with different colors.

Overall*, *the genomes of *B. longum* subsp. *longum* showed the highest number of accessory GH genes [Figure 2 and Supplementary Table 3]. Indeed, within the chromosomes of this taxon, 24 GH families were found in 9%-90% of the strains, in comparison of only 2-8 GH families constituting the accessory GH arsenal of the other considered probiotic (sub)species [Figure 2 and Supplementary Table 3]. In particular, GH families involved in the degradation of HMOs and host glycan structures, i.e., GH129, GH136, GH85 (endo-β-N-acetylglucosaminidase), and GH29, were found in, respectively, 72.7%, 63.6%, 45.5%, and 9.1% of the probiotic strains belonging to *B. longum* subsp. *longum*.

Although carbohydrate utilization capabilities are often associated with (sub)species-specific features, IPDB analyses reported differences in carbohydrate-metabolizing enzymes between commercialized probiotics of the same (sub)species. Such differences can have functional and ecological implications worthy of consideration for probiotic formulation and consumption.

### Extracellular structures involved in microbe-host interactions

Bacterial extracellular appendages, such as pili or fimbriae, are long and non-flagellar structures strategically localized to the cell surface to promote bacterial adhesion in the gut, simultaneously impacting microbe-host dialogue^[38,39]^. In the *Bifidobacterium *genus, SD pili (types I and II), collectively representing the SD fimbriome, as well as type IV pili, have been previously described^[26]^. While these latter are highly conserved among bifidobacterial genomes, the SD pili showed a considerable variability^[40]^. Thus, we explored the SD pili-encoding genes arsenal of the 34 collected bifidobacterial probiotic strains exploiting a custom database built in the contest of a previous study^[26]^.

**Figure 3:**
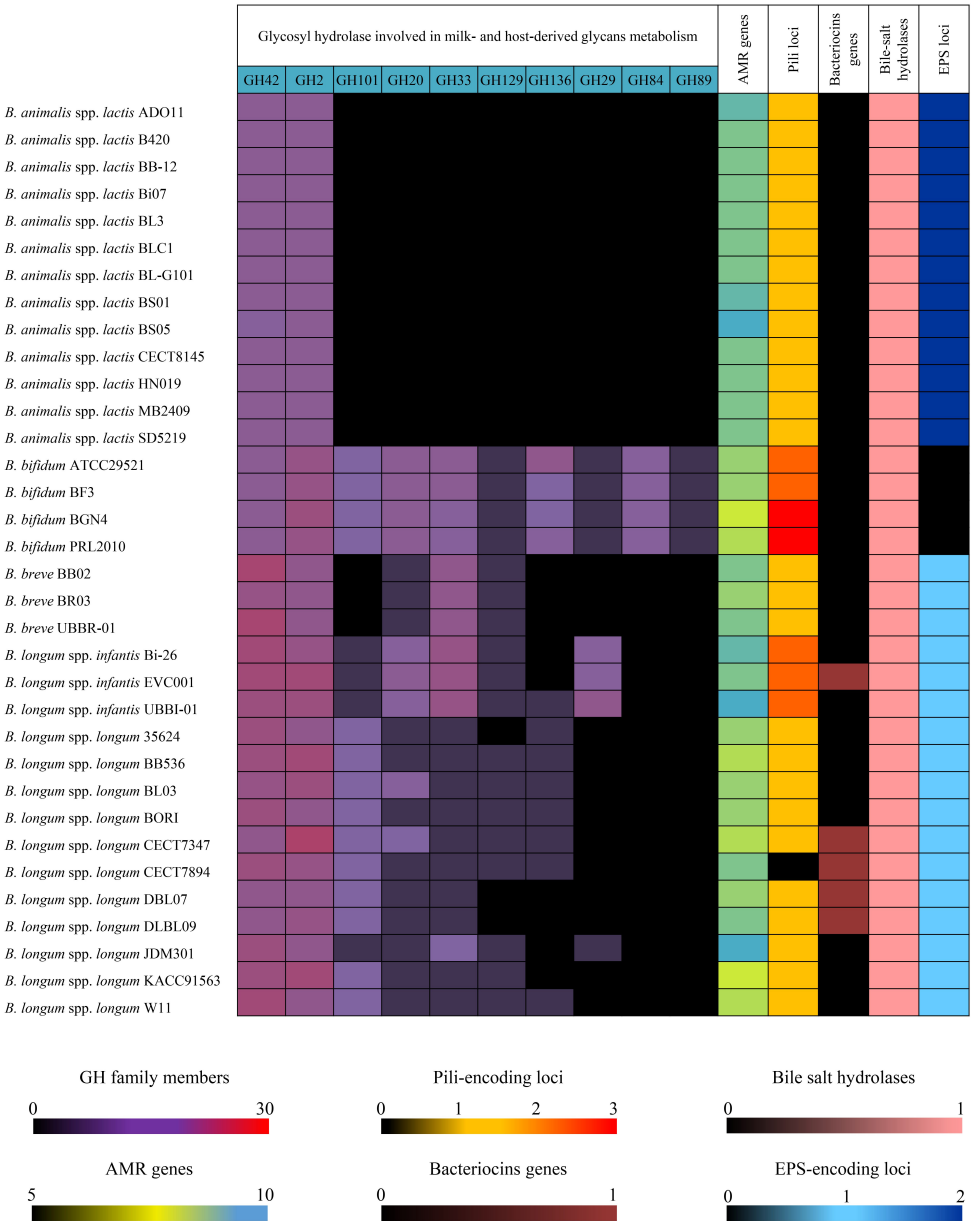
Occurrence of genetic probiotic features in the bifidobacterial strains. For each of the 34 considered bifidobacterial probiotic strain, the heat map shows the predicted number of glycosyl hydrolase enzymes involved in host glycan metabolisms, antimicrobial resistance determinants, pili- and bacteriocins-encoding genes, bile salt hydrolases, and exopolysaccharides (EPSs)-encoding loci.

Overall, SD pilus gene clusters, composed of a sortase-encoding gene for assembling pilus subunits and two pilin subunit-encoding genes, were found in 91% of the inspecting genomes [Figure 3 and Supplementary Table 4]. Interestingly, while genome sequences of *B. longum* susp. *infantis *appear unable to encode this type of pili, probiotic strains belonging to *B. bifidum* possessed the highest number of SD pili-encoding clusters [Figure 3 and Supplementary Table 4]. In particular, the genomes of *B. bifidum* strains BGN4 and PRL2010 showed three SD pili loci, thus suggesting putative improved adherence and persistence features. Furthermore, a diverse array of genes required for the production of SD pili was observed between probiotic strains belonging to the same (sub)species. In particular, *B. longum* subsp. *longum* showed a variable number of SD pili, ranging 0-2 [Figure 3 and Supplementary Table 4], highlighting possible different abilities to colonize and persist in the human gastrointestinal tract.

Overall, these data collected in the IPDB, in addition to (sub)species-specific features, highlight considerable strain-level variabilities in the environment interaction structures that could therefore determine different individual probiotic properties.

### Production of bacteriocins by commercial bifidobacterial probiotics

In addition to external structures, bifidobacteria exploit molecule-based systems to compete for intestinal colonization directly. Although the inhibitory activity of bifidobacteria could partially derive from the production of organic acids, it is hypothesized that some members of the *Bifidobacterium* genus can produce antimicrobial molecules such as bacteriocins^[41,42]^. These latter are ribosomally synthesized peptides with antimicrobial activities against other bacteria, either belonging to the same species or even across genera^[43,44]^. Consequently, these compounds are regarded as a probiotic trait contributing to higher niche competitiveness and inhibition of intestinal pathogens^[45]^. For this reason, we investigated the occurrence of bacteriocins-encoding genes among the 34 bifidobacterial probiotics using the BAGEL4 database^[27]^.

As a result, five potential bacteriocin genes were predicted to be codified by *B. longum* subsp. *infantis *and *B. longum* subsp. *longum *probiotic strains. In particular, a Class I lantibiotic (BLD_1648) was found in *B. longum* subsp. *infantis* EVC001 and in four members of *B. longum* subsp. *longum* taxon, i.e., strains CECT7347, CECT7894, DLBL07, and DLBL09 [Figure 3 and Supplementary Table 4].

Based on these *in silico* analyses results, only a limited number of *Bifidobacterium* species encode for bacteriocins, and intra-(sub)species variabilities have been found when comparing different strains. In particular, only (certain) strains belonging to *B. longum* subsp. *longum* and *B. longum* subsp. *infantis* showed strain-specific abilities in producing antimicrobial compounds, which may facilitate the introduction of the (probiotic) producer into an established niche by directly inhibiting competing strains or pathogens. Thus, these findings evidenced by analysis of the IPDB reinforce the need for a precise assessment of desirable probiotic properties, such as bacteriocins production, at a strain-specific level.

### Antibiotics resistance prediction and their distribution among commercial bifidobacterial probiotics

Probiotics are specifically selected not to carry AMR, with particular attention to AMR determinants located in the proximity of transposable elements or falling inside (integrated) bacterial plasmids, which could contribute to the spread of AMR^[46,47]^. Notably, AMR determinant surveys across the *Bifidobacterium* genus revealed that, except for tetracyclines resistance (*tet* genes) in specific cases, the resistance phenotypes are independent of the presence of particular genes, or they do not fall in genomic regions involved in horizontal gene transfer events. Hence, they rarely represent a risk for transfer to unrelated pathogenic or potentially pathogenic bacteria^[48,49]^. Conversely, AMR can enhance the survival of the probiotics in the presence of antimicrobial compounds due to medical treatments, thus constituting a beneficial feature^[50]^.

In this contest, the collected 34 bifidobacterial probiotic strains were inspected for putative antibiotic resistance determinants. Even if our *in-silico* analysis remains only predictive, such an approach can provide indications for further *in vitro* validations. As a result, an average of 7.6 AMR genes per chromosome were identified [Supplementary Table 5]. Among these, a putative ATP-binding cassette (ABC) transporter that exports macrolides (ARO:3000535) and putative rifampicin (ARO:3004480), fosfomycin (ARO:3003785), and mupirocin (ARO:3003730) resistances were found shared by all probiotics, while a gene conferring resistance to cationic antimicrobial peptides (ARO:3003577) was shared by 76% of bifidobacterial probiotics strains [Figure 3 and Supplementary Table 5]. Notably, prediction of the putative transporter specificity was assessed with manual validation employing the TCDB^[24]^.

In addition, (sub)species-specific AMR genes were also observed, including genes putatively involved in resistance against several different classes of antibiotics, i.e., multidrug efflux transporter, within *Blon*-CG (ARO:3000816) and *Bbre*-CG (ARO:3002813), and genes conferring putative resistance to mycinamicin (ARO:3001301) and tetracycline antibiotics (ARO:3003980, ARO:3000194) in *Blac*-CG [Figure 3 and Supplementary Table 5]. 

Focusing on the unique gene pools, which could be horizontally acquired, strain-specific AMR genes were found in 4 out of the 34 screened probiotic strains [Figure 3 and Supplementary Table 5]. In particular, *B. animalis* subsp. *lactis* BS05, showing the lowest ANI value, i.e., 99.3%, when compared to the other genomes of the same species, was predicted to encode a mosaic tetracycline resistance gene (*tetW/N/W*, ARO:3004442) and a gene possibly involved in resistances to carbapenems, rifamycin, and peptide antibiotics (ARO:3005059) [Figure 3 and Supplementary Table 5]. Moreover, two different macrolide resistance systems (ARO:3000616 and ARO:3004626) were noticed in the *B. longum* subsp. *longum* CECT7894 and JDM301 strains, respectively, whereas a generic antibiotic efflux pump (ARO:3000838) was observed in the strain DLBL09 [Figure 3 and Supplementary Table 5].

Based on the data collected in the IPDB, bifidobacterial probiotics appear to possess a relatively low acquired resistance compared to members of the *Enterococcus* and *Lactobacillus* genus used as probiotics in humans and farm animals^[51]^, for which resistance to a wide range of antimicrobials carried on plasmids or in the proximity of conjugative transposons has been identified^[52-54]^. Nevertheless, strain-specific AMR determinants have been observed, highlighting the need for case-by-case assessments. 

### Screening of additional genetic features involved in colonization and persistence

To further characterize the 34 bifidobacterial probiotic strains included in the database, attributes including bile salt tolerance mediated by bile salt hydrolases, production of exopolysaccharides (EPSs), and the presence of putative virulence factors were investigated. Notably, the ability to hydrolyze bile salts is often regarded as a desirable feature for probiotic strain selection since it can promote probiotic fitness and colonization by detoxifying bile^[55]^. According to *in silico* analyses, bile salt hydrolase activity has been predicted for all 34 bifidobacterial strains [Supplementary Table 6], resulting to be a widespread trait among the bifidobacterial probiotics. Furthermore, screening of potential virulence-related genes revealed the presence of homologous genes associated to surface carbohydrates polymers and response regulator proteins which typically mediate the interaction with the surrounding environment [Supplementary Table 7]. However, such structures are not recognized as harmful. Instead, they are well-known to participate in the host-microbe dialogue underlying and supporting the claimed probiotic effects^[56]^. Consistently, the analysis revealed the absence of genes associated with clear detrimental effects, remarking the safe use of bifidobacterial strains as probiotics.

Among the interesting and attractive characteristics of probiotic strains, the production of EPSs has grasped the attention because of its important role in maintaining commensalism between human host and (bifido)bacteria as well as for their putative health-promoting properties^[57,58]^. EPSs are extracellular carbohydrate polymers, and, for their biosynthesis, a gene cluster including a pGTF and additional genes, such as ABC transporters, subunit polymerization enzymes, and carbohydrate precursor biosynthesis/modification enzymes, are required^[59]^. In particular, pGTF is an essential enzyme that catalyzes the first step of the EPS biosynthetic pathway^[59]^.

In this context, the 34 bifidobacterial probiotics were explored for EPS loci employing well-known pGTF gene sequences as molecular indicators, as previously performed^[60,61]^.

Accordingly, the production of EPSs was predicted in all bifidobacterial chromosomes except for those belonging to *B. bifidum* species. Specifically, the presence of two highly conserved EPS loci were observed in each *B. animalis* subsp. *lactis *probiotic [Supplementary Table 6], while a single EPS-producing locus with a significant intra-(sub)species variability was detected among probiotic strains belonging to *B. longum* subsp. *longum* and *B. longum* subsp. *infantis* taxa [Supplementary Table 6]. The precise location of the pGTFs predicted in each bifidobacterial genome is reported in Supplementary Table 6.

## CONCLUSION

Because of their safety, functional, and technological characteristics, various members of the *Bifidobacterium* genus have been commercially available to and steadily used as probiotic bacteria.

In this study, we constructed the first iteration of a genomic database named IPDB encompassing 34 publicly available strains of *B. bifidum*, *B. longum* subsp. *longum*, *B. longum* subsp. *infantis*, *B. breve*, and *B. animalis* subsp. *lactis* (sub)species used in commercialized health-promoting supplements. The collected genome sequences were re-analyzed using an updated bioinformatics pipeline, and all the acquired genetic and functional information was included in the IPDB. Comparative genome analyses, in addition to genetic determinants shared by all the members of a species, revealed the existence of a range of strain-unique features possibly related to probiotic activities.

In particular, the greater number of host glycans-metabolizing and pili-encoding genes found in the genome sequences of *B. bifidum* and *B. longum* subsp. *infantis* (sub)species reflect their higher capability to colonize and persist in the human gastrointestinal tract as well as in those of lactating infants. On the other hand, strain-specific host-derived glycans metabolic machinery was deployed by some strains of *B. longum* subsp. *longum*, reflecting intra-(sub)species differences in enhancing digestion and absorption of nutrients in breastfed infants. Moreover, strain-dependent differences in bacteriocins production, EPSs biosynthesis, and antibiotic resistance were noticed not only among probiotic species but potentially among strains of the same species. Accordingly, strain-specific gene arsenals deserve attention since they can be correlated with profoundly different ecological behavior in the intestinal environment and the dialogue with the host, thus leading to different probiotic outcomes. As a result, accurate strain-level information about probiotic products should now be considered necessary to allow consumers to obtain precise evidence behind the claimed beneficial effects of each probiotic.

In this context, the IPDB represents a novel, intriguing instrument to rapidly access the genome content of common bifidobacterial probiotic strains, assisting in drawing the connection among probiotics, gut microbiome, and beneficial effects to the host.

## DECLARATIONS

### Availability of data and materials

The IPDB can be accessed at http://probiogenomics.unipr.it/cmu/, (direct download at http://probiogenomics.unipr.it/files/Probiotic_Bifidobacteria_DataBase).

### Copyright

© The Author(s) 2022.

## References

[1] Hill C, Guarner F, Reid G (2014). Expert consensus document. The International Scientific Association for Probiotics and Prebiotics consensus statement on the scope and appropriate use of the term probiotic. Nat Rev Gastroenterol Hepatol.

[2] Aditya A, Peng M, Young A, Biswas D (2020). Antagonistic mechanism of metabolites produced by lactobacillus casei on lysis of enterohemorrhagic escherichia coli. Front Microbiol.

[3] Tomasik PJ, Tomasik P (2003). Probiotics and prebiotics. Cereal Chemistry.

[4] Koutsoumanis K, Allende A, Alvarez-Ordóñez A, EFSA Panel on Biological Hazards (BIOHAZ) (2021). Update of the list of QPS-recommended biological agents intentionally added to food or feed as notified to EFSA 14: suitability of taxonomic units notified to EFSA until March 2021. EFSA J.

[5] Scheinbach S (1998). Probiotics: functionality and commercial status. Biotechnology Advances.

[6] Papizadeh M, Rohani M, Nahrevanian H, Javadi A, Pourshafie MR (2017). Probiotic characters of Bifidobacterium and Lactobacillus are a result of the ongoing gene acquisition and genome minimization evolutionary trends. Microb Pathog.

[7] McFarland LV (2021). Efficacy of single-strain probiotics versus multi-strain mixtures: systematic review of strain and disease specificity. Dig Dis Sci.

[8] Ouwehand AC, Invernici MM, Furlaneto FAC, Messora MR (2018). Effectiveness of multistrain versus single-strain probiotics: current status and recommendations for the future. J Clin Gastroenterol.

[9] Lugli GA, Milani C, Mancabelli L, van Sinderen D, Ventura M (2016). MEGAnnotator: a user-friendly pipeline for microbial genomes assembly and annotation. FEMS Microbiol Lett.

[10] Hyatt D, Chen GL, Locascio PF, Land ML, Larimer FW, Hauser LJ (2010). Prodigal: prokaryotic gene recognition and translation initiation site identification. BMC Bioinformatics.

[11] Zhao Y, Tang H, Ye Y (2012). RAPSearch2: a fast and memory-efficient protein similarity search tool for next-generation sequencing data. Bioinformatics.

[12] Lowe TM, Eddy SR (1997). tRNAscan-SE: a program for improved detection of transfer RNA genes in genomic sequence. Nucleic Acids Res.

[13] Lagesen K, Hallin P, Rødland EA, Staerfeldt HH, Rognes T, Ussery DW (2007). RNAmmer: consistent and rapid annotation of ribosomal RNA genes. Nucleic Acids Res.

[14] Zhao Y, Wu J, Yang J, Sun S, Xiao J, Yu J (2012). PGAP: pan-genomes analysis pipeline. Bioinformatics.

[15] Enright AJ, Van Dongen S, Ouzounis CA (2002). An efficient algorithm for large-scale detection of protein families. Nucleic Acids Res.

[16] Huerta-Cepas J, Szklarczyk D, Forslund K (2016). eggNOG 4.5: a hierarchical orthology framework with improved functional annotations for eukaryotic, prokaryotic and viral sequences. Nucleic Acids Res.

[17] Jain C, Rodriguez-R LM, Phillippy AM, Konstantinidis KT, Aluru S (2018). High throughput ANI analysis of 90K prokaryotic genomes reveals clear species boundaries. Nat Commun.

[18] Katoh K, Misawa K, Kuma K, Miyata T (2002). MAFFT: a novel method for rapid multiple sequence alignment based on fast Fourier transform. Nucleic Acids Res.

[19] Chenna R, Sugawara H, Koike T (2003). Multiple sequence alignment with the Clustal series of programs. Nucleic Acids Res.

[20] Lombard V, Golaconda Ramulu H, Drula E, Coutinho PM, Henrissat B (2014). The carbohydrate-active enzymes database (CAZy) in 2013. Nucleic Acids Res.

[21] Wheeler TJ, Eddy SR (2013). nhmmer: DNA homology search with profile HMMs. Bioinformatics.

[22] Altschul SF, Gish W, Miller W, Myers EW, Lipman DJ (1990). Basic local alignment search tool. J Mol Biol.

[23] Alcock BP, Raphenya AR, Lau TTY (2020). CARD 2020: antibiotic resistome surveillance with the comprehensive antibiotic resistance database. Nucleic Acids Res.

[24] Saier MH Jr, Reddy VS, Tsu BV, Ahmed MS, Li C, Moreno-Hagelsieb G (2016). The Transporter Classification Database (TCDB): recent advances. Nucleic Acids Res.

[25] Ye Y, Choi JH, Tang H (2011). RAPSearch: a fast protein similarity search tool for short reads. BMC Bioinformatics.

[26] Milani C, Mangifesta M, Mancabelli L (2017). The Sortase-dependent fimbriome of the genus bifidobacterium: extracellular structures with potential to modulate microbe-host dialogue. Appl Environ Microbiol.

[27] (2018). Heel AJ, de Jong A, Song C, Viel JH, Kok J, Kuipers OP. BAGEL4: a user-friendly web server to thoroughly mine RiPPs and bacteriocins. Nucleic Acids Res.

[28] Chen L, Yang J, Yu J (2005). VFDB: a reference database for bacterial virulence factors. Nucleic Acids Res.

[29] Tarracchini C, Milani C, Lugli GA (2021). Phylogenomic disentangling of the Bifidobacterium longum subsp. infantis taxon. Microb Genom.

[30] Blanco-Míguez A, Gutiérrez-Jácome A, Fdez-Riverola F, Lourenço A, Sánchez B (2016). A peptidome-based phylogeny pipeline reveals differential peptides at the strain level within Bifidobacterium animalis subsp. lactis. Food Microbiol.

[31] Underwood MA, German JB, Lebrilla CB, Mills DA (2015). Bifidobacterium longum subspecies infantis: champion colonizer of the infant gut. Pediatr Res.

[32] Tan J, Mckenzie C, Potamitis M, Thorburn AN, Mackay CR, Macia L

[33] Abdelhamid AG, El-Dougdoug NK (2021). Comparative genomics of the gut commensal Bifidobacterium bifidum reveals adaptation to carbohydrate utilization. Biochem Biophys Res Commun.

[34] Sela DA, Garrido D, Lerno L (2012). Bifidobacterium longum subsp. infantis ATCC 15697 α-fucosidases are active on fucosylated human milk oligosaccharides. Appl Environ Microbiol.

[35] Katoh T, Ojima MN, Sakanaka M, Ashida H, Gotoh A, Katayama T (2020). Enzymatic adaptation of bifidobacterium bifidum to Host Glycans, viewed from glycoside hydrolyases and carbohydrate-binding modules. Microorganisms.

[36] Sakurama H, Kiyohara M, Wada J (2013). Lacto-N-biosidase encoded by a novel gene of Bifidobacterium longum subspecies longum shows unique substrate specificity and requires a designated chaperone for its active expression. J Biol Chem.

[37] Odamaki T, Bottacini F, Kato K (2018). Genomic diversity and distribution of Bifidobacterium longum subsp. longum across the human lifespan. Sci Rep.

[38] Turroni F, Serafini F, Foroni E (2013). Role of sortase-dependent pili of Bifidobacterium bifidum PRL2010 in modulating bacterium-host interactions. Proc Natl Acad Sci U S A.

[39] Nishiyama K, Yokoi T, Sugiyama M, Osawa R, Mukai T, Okada N (2021). Roles of the cell surface architecture of Bacteroides and Bifidobacterium in the gut colonization. Front Microbiol.

[40] Alessandri G, van Sinderen D, Ventura M (2021). The genus bifidobacterium: from genomics to functionality of an important component of the mammalian gut microbiota running title: bifidobacterial adaptation to and interaction with the host. Comput Struct Biotechnol J.

[41] Martinez FA, Balciunas EM, Converti A, Cotter PD, de Souza Oliveira RP (2013). Bacteriocin production by Bifidobacterium spp. A review. Biotechnol Adv.

[42] Niederhäusern S, Camellini S, Sabia C, Iseppi R, Bondi M, Messi P (2020). Antilisterial activity of bacteriocins produced by lactic bacteria isolated from dairy products. Foods.

[43] Kanmani P, Satish Kumar R, Yuvaraj N, Paari KA, Pattukumar V, Arul V (2013). Probiotics and its functionally valuable products-a review. Crit Rev Food Sci Nutr.

[44] Liévin V, Peiffer I, Hudault S (2000). Bifidobacterium strains from resident infant human gastrointestinal microflora exert antimicrobial activity. Gut.

[45] O'Shea EF, Cotter PD, Stanton C, Ross RP, Hill C (2012). Production of bioactive substances by intestinal bacteria as a basis for explaining probiotic mechanisms: bacteriocins and conjugated linoleic acid. Int J Food Microbiol.

[46] Gama JA, Zilhão R, Dionisio F (2018). Impact of plasmid interactions with the chromosome and other plasmids on the spread of antibiotic resistance. Plasmid.

[47] Lupski JR (1987). Molecular mechanisms for transposition of drug-resistance genes and other movable genetic elements. Rev Infect Dis.

[48] Kiwaki M, Sato T (2009). Antimicrobial susceptibility of Bifidobacterium breve strains and genetic analysis of streptomycin resistance of probiotic B. breve strain Yakult. Int J Food Microbiol.

[49] Sato T, Iino T (2010). Genetic analyses of the antibiotic resistance of Bifidobacterium bifidum strain Yakult YIT 4007. Int J Food Microbiol.

[50] Gueimonde M, Sánchez B, G de Los Reyes-Gavilán C, Margolles A (2013). Antibiotic resistance in probiotic bacteria. Front Microbiol.

[51] Franz CM, Huch M, Abriouel H, Holzapfel W, Gálvez A (2011). Enterococci as probiotics and their implications in food safety. Int J Food Microbiol.

[52] Miller WR, Munita JM, Arias CA (2014). Mechanisms of antibiotic resistance in enterococci. Expert Rev Anti Infect Ther.

[53] Patel R (2000). Enterococcal-type glycopeptide resistance genes in non-enterococcal organisms. FEMS Microbiol Lett.

[54] Vescovo M, Morelli L, Bottazzi V (1982). Drug resistance plasmids in Lactobacillus acidophilus and Lactobacillus reuteri. Appl Environ Microbiol.

[55] Jarocki P, Podleśny M, Glibowski P, Targoński Z (2014). A new insight into the physiological role of bile salt hydrolase among intestinal bacteria from the genus Bifidobacterium. PLoS One.

[56] Castro-Bravo N, Wells JM, Margolles A, Ruas-Madiedo P (2018). Interactions of surface exopolysaccharides from Bifidobacterium and Lactobacillus within the intestinal environment. Front Microbiol.

[57] Prasanna P, Grandison A, Charalampopoulos D (2014). Bifidobacteria in milk products: an overview of physiological and biochemical properties, exopolysaccharide production, selection criteria of milk products and health benefits. Food Res Int.

[58] Fanning S, Hall LJ, Cronin M (2012). Bifidobacterial surface-exopolysaccharide facilitates commensal-host interaction through immune modulation and pathogen protection. Proc Natl Acad Sci U S A.

[59] Provencher C, LaPointe G, Sirois S, Van Calsteren MR, Roy D (2003). Consensus-degenerate hybrid oligonucleotide primers for amplification of priming glycosyltransferase genes of the exopolysaccharide locus in strains of the Lactobacillus casei group. Appl Environ Microbiol.

[60] Ferrario C, Milani C, Mancabelli L (2016). Modulation of the eps-ome transcription of bifidobacteria through simulation of human intestinal environment. FEMS Microbiol Ecol.

[61] Yan S, Zhao G, Liu X, Zhao J, Zhang H, Chen W (2017). Production of exopolysaccharide by Bifidobacterium longum isolated from elderly and infant feces and analysis of priming glycosyltransferase genes. RSC Adv.

