# Supplementary figures and images for: The Integrated Probiotic Database: a genomic compendium of bifidobacterial health-promoting strains
Source: Microbiome Res Rep. 2022 Feb 28;1(2):9. doi: 10.20517/mrr.2021.13 (PMC10688828; doi:10.20517/mrr.2021.13)

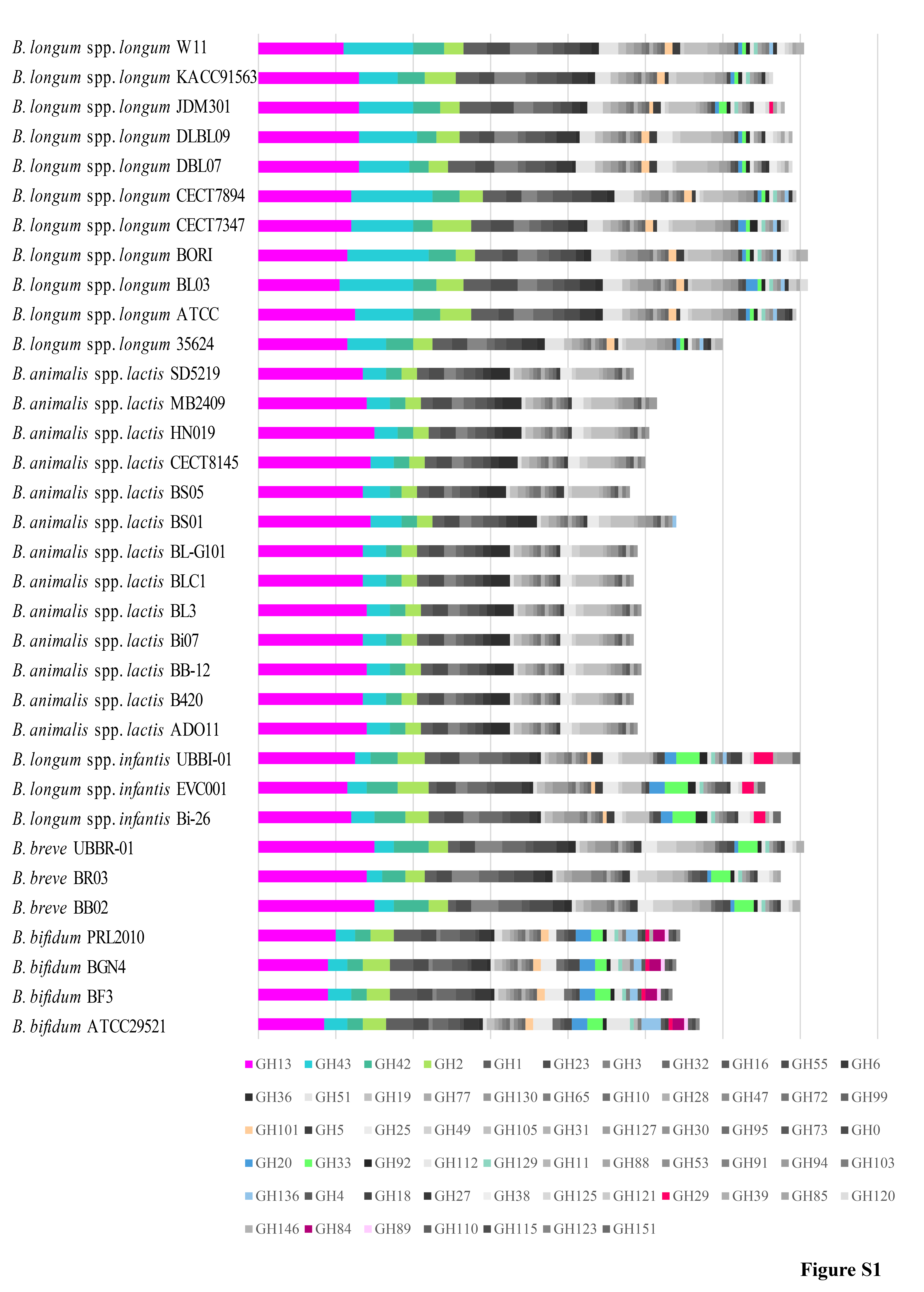

Supplement: Supplementary file 1 [file mrr-1-2-9-SupplementaryMaterials.zip › 202113-SupplementaryMaterials/Supplementary Figure S1.png]
